# Effects of *cis*-Jasmone Treatment of Brassicas on Interactions With *Myzus persicae* Aphids and Their Parasitoid *Diaeretiella rapae*

**DOI:** 10.3389/fpls.2021.711896

**Published:** 2021-10-01

**Authors:** Jamin Ali, Anca D. Covaci, Joe M. Roberts, Islam S. Sobhy, William D. J. Kirk, Toby J. A. Bruce

**Affiliations:** ^1^School of Life Sciences, Keele University, Keele, United Kingdom; ^2^Agriculture and Environment Department, Centre for Integrated Pest Management, Harper Adams University, Newport, United Kingdom; ^3^Department of Plant Protection, Faculty of Agriculture, Suez Canal University, Ismailia, Egypt

**Keywords:** induced defence, aphid, tritrophic interactions, biological control, crop protection

## Abstract

There is a need to develop new ways of protecting plants against aphid attack. Here, we investigated the effect of a plant defence activator, *cis*-jasmone (CJ), in a range of cultivars of *Brassica napus, Brassica rapa* and *Brassica oleracea*. Plants were sprayed with *cis*-jasmone or blank formulation and then tested with peach potato aphids (*Myzus persicae* Sulzer) (Hemiptera: Aphididae) and their parasitoid *Diaeretiella rapae* (M'Intosh) (Hymenoptera: Braconidae). CJ treated plants had significantly lower aphid settlement than control plants in a settlement bioassay. Conversely, in a foraging bioassay, *D. rapae* parasitoids spent a significantly longer time foraging on CJ treated plants. Our results reveal that CJ treatment makes plants less attractive to and less suitable for *M. persicae* but more attractive to *D. rapae* in a range of brassica cultivars. It is likely that these effects are due to changes in volatile emission indicating activation of defence and presence of conspecific competitors to aphids but presence of prey to parasitoids. Increases in volatile emission were found in CJ induced plants but varied with genotype. Among the synthetic volatile compounds that were induced in the headspace of CJ treated brassica cultivars, methyl isothiocyanate, methyl salicylate and *cis*-jasmone were most repellent to aphids. These results build on earlier studies in *Arabidopsis* and show that tritrophic interactions are influenced by CJ in a wide range of brassica germplasm. The implication is that CJ is a promising treatment that could be used in brassica crops as part of an integrated pest management system.

## Introduction

*cis-*Jasmone (CJ) is a benign plant defence activator shown to have considerable promise in enhancing plant defence against hemipteran insect pests in *Arabidopsis thaliana* (Bruce et al., 2008) as well as crops such as wheat (Bruce et al., 2003a,b), maize (Oluwafemi et al., 2013), cotton (Hegde et al., 2012), sweet pepper (Dewhirst et al., 2012) and potato (Sobhy et al., 2017, 2020). CJ was first tested and reported as a plant defence activator by Birkett et al. (2000) after the compound was found to be highly electrophysiologically active with aphids. It was hypothesised to have a role in plant defence due to structural similarities with the plant hormone jasmonic acid and experimental evidence confirmed this hypothesis. Genes activated by CJ are, however, different from the ones activated by jasmonic acid or methyl jasmonate (Matthes et al., 2010).

Basic studies done in *A. thaliana* demonstrated that CJ makes plants less attractive to aphids but more attractive to their parasitoid natural enemies (Bruce et al., 2008; Matthes et al., 2010). We hypothesised that similar effects would occur in brassica crops, but data on this were not previously available and there was a knowledge gap relating to this. Therefore, the current study was designed to test the hypotheses that CJ treatment could (1) reduce aphid performance and colonisation and (2) increase parasitoid foraging in a range of cultivars of *Brassica napus, B. rapa* and *B. oleracea*. Our study focused on *Myzus persicae* Sulzer (Hemiptera: Aphididae), commonly known as peach potato aphid, because it is the main aphid pest of brassica crops (van Emden et al., 1969). *Diaeretiella rapae* (M'Intosh) (Hymenoptera: Braconidae) was chosen as the parasitoid species because a survey conducted by ADAS revealed it was the most common parasitoid of *M. persicae* in the UK (Jude Bennison, *pers. comm*.).

Our study is timely because there is an urgent need for new approaches to crop protection in brassicas as *Myzus persicae* aphids have evolved resistance to many insecticides (Bass et al., 2014) and the neonicotinoid restriction in Europe has further reduced conventional control options (Dewar, 2017).

## Materials and Methods

### Insects

*Myzus persicae* aphids (clone O) were reared on pak choi (*Brassica rapa* subsp. *chinensis* cv. Hanakan) in BugDorm cages (46 × 46 × 46 cm; NHBS Ltd, Devon, UK) under controlled environmental conditions (24°C, 38 % RH, 16:8 photoperiod) in the Centre of Applied Entomology and Parasitology (CAEP) insectary at Keele University (UK). *Diaeretiella rapae* parasitoid wasps were reared on surplus *M. persicae* infested pak choi plants in BugDorms (46 × 46 × 46 cm) under controlled conditions (20°C, 40% RH, 16:8 photoperiod) in a growth chamber (MLR-352-PE; Panasonic, The Netherlands). Both cultures were originally obtained from Harper Adams University, UK.

### Plants

Six brassica cultivars, representing a wide range of germplasm, from three species were grown for use in experiments: *Brassica napus* cultivars Samurai, Wesway, English Giant and Turnip Rutabaga 57, *Brassica oleracea* cv. Warwick L9 (Chinese kale) and *Brassica rapa* subsp. *chinensis* cv. Hanakan (pak choi) were obtained from Warwick University, UK. The effect of CJ treatment has not been investigated previously in these cultivars. All plants were individually grown in 7.5 cm pots filled with John Innes No. 2 compost (Westland Horticulture Limited, Tyrone, UK) under controlled environment conditions (20°C, 37 % RH, 16:8 photoperiod) in a growth chamber (MLR-352-PE; Panasonic). Plants at BBCH growth stage 14 (i.e., five true leaves) were used for all experiments (Meier, 1997).

### Chemicals and *cis*-Jasmone Treatment

Chemical standards tested individually in olfactometer bioassays were *cis*-3-hexenyl acetate (≥98%), methyl salicylate (≥99%), *cis*-jasmone (≥97%), methyl isothiocyanate (97%), benzyl nitrile (98%), limonene (96%), nonanal (≥98%), *trans*-β-farnesene (90 %), and β-elemene (≥98%). Concentrations of 100 ng/μl of each synthetic volatile eluted in hexane (≥95%) were prepared. All standards including hexane were purchased from Sigma Aldrich (Gillingham, UK).

Plants were sprayed with an aqueous emulsion of *cis*-jasmone (CJ) (Sigma Aldrich, Buchs, Switzerland) as described in Bruce et al. (2003a). A stock CJ emulsion was formulated by mixing 25 μl of CJ with 100 μl of Tween 80 (Sigma Aldrich) in 100 ml of deionized water, while a blank formulation to act as a control was formulated by mixing 100 μl of Tween 80 in 100 ml of deionised water. Spray treatment was carried out using an Oshide spray bottle (100 ml; Zhengzhou Xinrui Tongda Metal and Material Co., Ltd. Henan Sheng, China) by applying three trigger pulls of spray formulation (250 μl) to each plant at a distance of 30 cm. Sprayed plants were left for 24 h and then used for experiments. Control plants and CJ treated plants were placed in different compartments to avoid any plant-plant interaction.

### Aphid Performance Clip-Cage Bioassay

Performance of *M. persicae* was assessed on the different brassica cultivars. There were two separate series of experiments with different plants: the first series recorded observations after 48 h and the second recorded observations after 96 h. Fresh plants and aphids were used in each replicate observation in each experiment. In each replicate, 10 adult alate *M. persicae* were placed in a clip cage (2.5 cm diameter, Bioquip Products Inc., USA), which was attached to the lower surface of plant leaves (Sobhy et al., 2020). Two clip cages were placed on each plant. Ten replicates (control and CJ treated) were performed for each cultivar. To assess the survival and fecundity of aphids, plants were left undisturbed in a controlled environment room (25°C, 37% RH, 16:8 photoperiod). Plants were assessed after 48 h (series 1) or 96 h (series 2). For assessment, leaves containing the cages were cut and cages were removed without losing any aphids. Numbers of live adults and newly larviposited nymphs were recorded.

### Aphid Settlement Bioassay

In this choice test bioassay, CJ treated and control plants were placed in a BugDorm insect cage (60 × 60 × 60 cm; NHBS Ltd, Devon, UK) and were kept in a controlled environment room (25°C, 37% RH, 16:8 photoperiod). Each BugDorm contained four plants (two treated and two control) at alternate positions. A vial containing 50 alate *M. persicae* was positioned in the centre of the cage, opened and then the cage was left for 24 h. Counts of settled aphids were recorded 24 h after release. Ten replicates were done for each cultivar. The position of treatments was alternated between replicates.

### Parasitoid Foraging Bioassay

To test CJ effect on parasitoids, the foraging behaviour of *D. rapae* females was monitored. Noldus Observer 4.1 software was used to record the behavioural observations. In an open-fronted cage, a single female parasitoid was released from a vial onto a plant leaf and then its foraging behaviour was monitored by direct observation. Time spent walking, still, and cleaning was recorded, as well as total time spent before the parasitoid left the plant. An observation was terminated when a parasitoid flew away from the plant, which was considered as leaving the foraging “patch.” Ten replicates of CJ treated and control plants were done for each cultivar. Treated and control replicates were observed alternately. All experiments were done between 9:00 a.m. and 2:00 p.m.

### Parasitism Bioassay

Effect of CJ on the parasitism rate of *D. rapae* was assessed in BugDorm (60 × 60 × 60 cm) cages as described above. Bioassays were repeated 10 times on different experimental days. Each plant was infested with 50 adult *M. persicae* that were released 2 h prior to the release of *D. rapae*. Eight female parasitoids were released into the cage following the experimental procedure of Sun et al. (2020). After 24 h, parasitoid females were collected from the cages and plants were kept contained in bread bags while aphid mummies developed. Experiments were conducted in a controlled environment room (20°C, 37% RH, 16:8 photoperiod). After 15 days, the number of mummified aphids on the treated and control plants was recorded.

### Volatile Collection

Plant volatiles were collected following a procedure adapted from Agelopoulos et al. (1999) in which whole plants were contained inside oven bags (35 × 43 cm; Bacofoil, UK). The bag was partially sealed, so that only volatiles produced by the plants were collected. Prior to entrainments, bags were baked in an oven (Heraeus, Thermo Electron corporation, Mark Biosciences, UK) at 120°C overnight. Porapak Q philtres (0.05 g, 60/80 mesh; Supelco, Bellefonte, PA, USA) were washed with diethyl ether and then conditioned before use. Plants were enclosed in bags individually. Each bag was open at the bottom and closed at the top. An outlet hole was made in the upper part of the bag to connect the Porapak Q filter, whereas the bag bottom was closed by attaching a rubber band around the pot. Charcoal filtered air was pumped into bags at 600 ml min^−1^ and sampled air was pulled out at 400 ml min^−1^ through a Porapak Q filter in which the plant volatiles were trapped. To avoid entry of unfiltered air, positive pressure was maintained using differing air flows rates. Connections were made with 1.6 mm (i.d.) polytetrafluoroethylene (PTFE) tubing (Alltech Associates Inc., Lancashire, UK) with Swagelok brass ferrules and fittings (North London Valve Co., London, UK) and sealed with PTFE tape (Gibbs & Dandy Ltd., Luton, UK). Volatile collection was done for a 48 h period, after which the Porapak filters were eluted with 500 μl of diethyl ether into sample vials (Supelco, 2 ml, PTFE/silicone) and stored at −20°C in a freezer (Lec Medical, UK) for use in olfactometer bioassays and chemical analysis.

### Olfactometer Bioassay

The behavioural responses of alate *M. persicae* to brassica volatiles were investigated using a Perspex 4-arm olfactometer in a controlled environment room (24°C, 30% RH). The central area at the top of the olfactometer contained a hole into which a single aphid was introduced, and which was connected to a low-pressure air pump to remove air at a rate of 200 ml min^−1^. PTFE tape was used to ensure airtight seals between the olfactometer and the Teflon tubing. Holes connected to odour source tubes were covered with a layer of muslin to prevent aphid escape during the bioassays. The olfactometer arena was split into five areas; four areas by each arm (one treatment arm vs. three control arms or two treatment and two control arms) and a central area (Webster et al., 2010). Adult aphids were collected from rearing cages in a separate insectary room and transferred to the olfactometer laboratory for acclimatisation and starved for 2 h before each trial. Individual aphids were added using a 000 paintbrush. Each aphid was exposed to a test sample for 12 min, and after every 3 min the position of the olfactometer was rotated clockwise by 90° to eliminate directional bias. Time spent in each arm was recorded using a software program (OLFA, F. Nazzi, Udine, Italy). Ten replicates were done for each comparison. For each experiment, filter paper (Whatman No. 1, Buckinghamshire, UK) strips (cut to 5 × 20 mm) were treated with an aliquot (10 μl) of the volatile sample treatment or solvent control, applied using a micropipette (Drummond “microcaps”; Drummond Scientific Co., USA), and allowed to evaporate for 30 s before placing in odour source tubes. If an aphid remained motionless for the first 2 min of a replicate it was recorded as unresponsive and excluded from analysis. All bioassays were performed between 10:00 a.m. and 1:00 p.m.

#### Headspace Samples of Plant Naturally Emitted Plant Volatile Blends

Three series of experiments were conducted with the natural volatile blends collected from plants: (series 1) volatiles from control plants (treated with blank formulation of water + tween) vs. solvent control (diethyl ether), (series 2) volatiles from CJ treated plants (CJ + tween + water) vs. solvent control (diethyl ether), and (series 3) a choice test with volatiles from control plants vs. CJ treated plants vs. solvent (diethyl ether). For the first two series experiments, one arm was assigned to the collected volatiles from plants whereas three control arms were treated similarly with the same volume of solvent (diethyl ether used for eluting the plant volatiles). In the third series experiment, the two treatments being compared were assigned to one arm each whereas the other two opposite arms were assigned to solvent control (diethyl ether).

#### Individual Synthetic Volatile Compounds

The behavioural responses of alate *M. persicae* to nine synthetic chemical compounds were investigated as described above. These volatiles were induced in the headspace of CJ treated brassica cultivars (see below). Each synthetic chemical compound was tested at a concentration of [100 ng/μl in hexane]. In these experiments, similar to series 1 and 2 described above, one arm was assigned to one synthetic volatile compound whereas three control arms were treated similarly with the same volume of solvent.

### Volatile Analysis

Analyses were carried out on a 7820A GC coupled to a 5977B single quad mass selective detector (Agilent Technologies, Cheadle, UK). The GC was fitted with a non-polar HP5-MS capillary column (30 m × 0.25 mm × 0.25 μm film thickness) coated with (5%-phenyl)-methylpolysiloxane (Agilent Technologies) and used hydrogen carrier gas at a constant flow rate of 1.2 ml/min. Automated injections of 1 μl were made using a G4513A autosampler (Agilent Technologies) in splitless mode (285°C), with oven temperature programmed from 35°C for 5 min then at 10°C/min to 285°C. Compounds were identified according to their mass spectrum, linear retention index relative to retention times of *n*-alkanes, and co-chromatography with authentic compounds.

### Statistical Analysis

#### Aphid Clip Cage Bioassay

Differences in the mean number of live aphids on control and CJ treated plants was compared for each brassica cultivar at two time-points (48 and 96 h) using generalised linear models (GLM) fitted with Poisson probability distributions. Differences in the mean number of aphid nymphs larviposited onto control and CJ treated plants were compared for each brassica cultivar at two time-points (48 and 96 h) using GLMs fitted with quasi-Poisson probability distributions to account for overdispersion. Plant treatment (i.e., control vs. CJ treated) was a fixed factor.

#### Aphid Settlement Bioassay

Differences in the mean number of aphids settling on control and CJ treated plants were compared for each brassica cultivar using GLMs with Poisson or quasi-Poisson probability distributions depending on dispersion. Plant treatment (i.e., control vs. CJ treated) was a fixed factor.

#### Parasitoid Foraging Bioassay

The total time spent by parasitoid wasps foraging on control and CJ treated plants was first analysed for each brassica cultivar using Shapiro-Wilk tests to determine whether the underlying data were Gaussian. As data for this bioassay was non-Gaussian, the response variable (i.e., time) was square root transformed and re-analysed using Shapiro-Wilk tests to confirm that transformed data were Gaussian. After transformation, differences in mean total parasitoid foraging time between control and CJ treated plants was evaluated for each brassica cultivar using two-sample *t*-tests.

#### Parasitism Bioassay

Differences in the mean number of mummified aphids on control and CJ treated plants were compared for each brassica cultivar using GLMs with Poisson or quasi-Poisson probability distributions depending on dispersion. Plant treatment (i.e., control vs. CJ treated) was a fixed factor.

#### Volatile Profiling

To visualise the overall differences in volatile profiles emitted from the six studied brassica cultivars, a principal component analysis (PCA) was performed using the concentrations of the detected volatiles as dependent variables. Loading and score plots were derived after mean-centering and log transformation of volatile data. Average linkage hierarchical clustering based on Ward clustering algorithm of the Euclidean distance measure for the differentially emitted VOCs was used to construct a heatmap displaying the concentrations of different volatiles. Visualisation, together with hierarchical clustering of VOC data, was done using the MetaboAnalyst online tool suite (Chong et al., 2018). Subsequently, univariate analyses of variances were performed to investigate whether the concentrations of individual volatile compounds differed with and without CJ treatment using SigmaPlot 12.3 (Systat Software Inc., USA).

#### Olfactometer Bioassays

The behavioural response of *M. persicae* was tested in two ways. For experiments with one treated arm vs. three solvent control treatments, data were analysed by a paired *t*-test. In this analysis, the time spent by aphids in treated and solvent arms of the four-arm olfactometer were compared. In experiments where the response in two treatment arms vs. two arms of solvent control was compared, data were first converted into proportions then log-ratio transformed before analysis by one-way analysis of variance and Holm-Sidak mean separation (Mwando et al., 2018). Data were examined for a Gaussian distribution using the Shapiro-Wilk test prior to analysis.

All statistical analyses were carried out using R (v 4.0.3) (R Core Development Team, 2021).

## Results

### Aphid Clip Cage Bioassay

**Figure 1:**
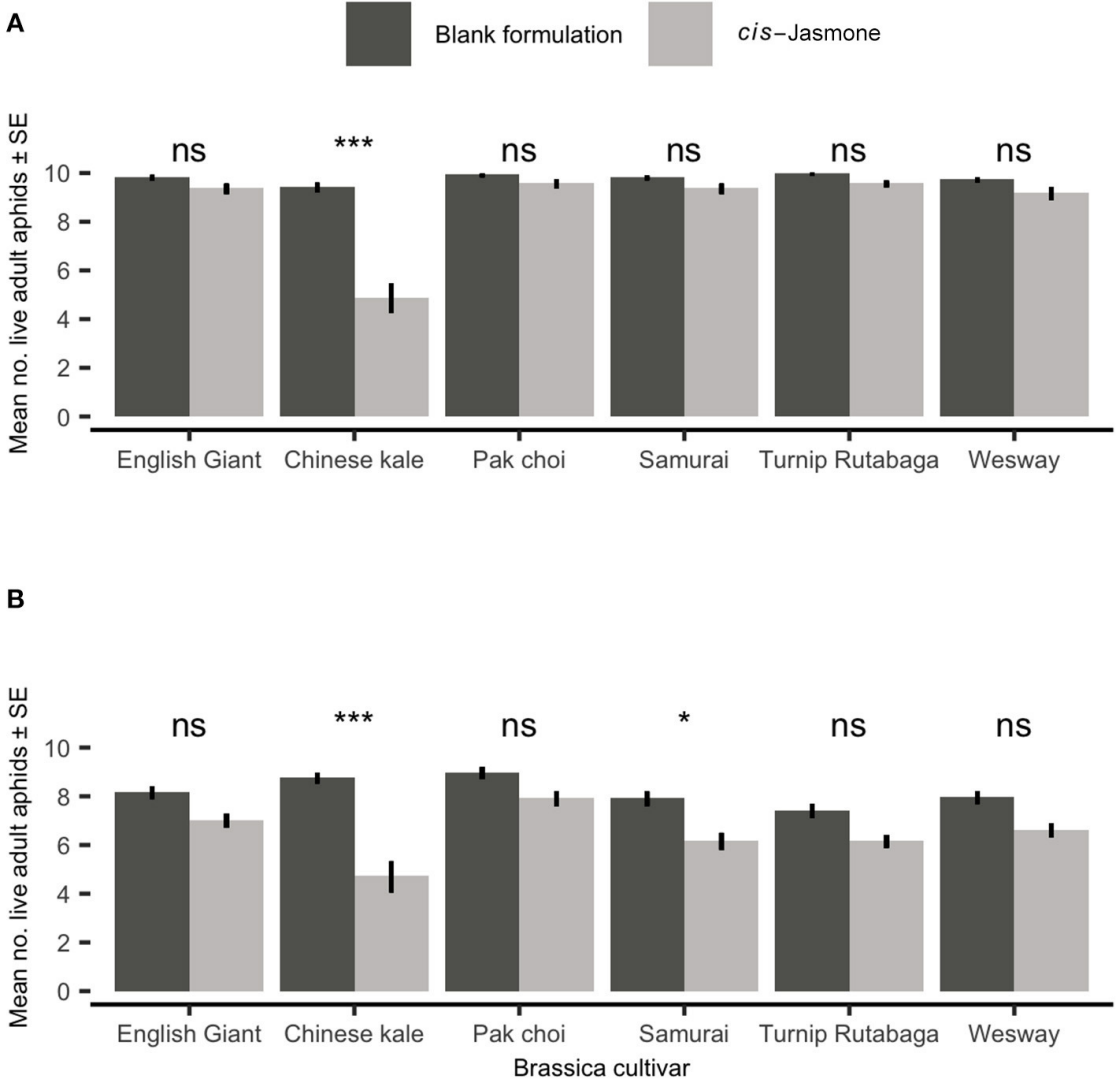
Adult *Myzus persicae* survival (Mean ± SE) out of the original 10 individuals after **(A)** 48 h and **(B)** 96 h in clip cages on six brassica cultivars (*n* = 10) treated with *cis-*jasmone or blank formulation (control). Brassica cultivars capped with “ns” do not show a significant difference between control and *cis*-jasmone treatment while asterisks denote differing levels of statistical significance: ^*^ < 0.05 and ^***^ < 0.001 (generalised linear models with Poisson probability distribution).

After 48 h there was no significant reduction in adult *M. persicae* survival on five brassica cultivars treated with CJ in clip cage experiments (Figure 1A). However, treating the Chinese kale cultivar with CJ reduced survival two-fold (generalised linear model with Poisson distribution: *X*^2^ = 12.50; *d.f*. = 1,38; *P* = 0.04). There was no significant reduction in adult *M. persicae* survival on four brassica cultivars treated with CJ in clip cage experiments after 96 h (Figure 1B). Both the Chinese kale (generalised linear model with Poisson distribution: *X*^2^ = 40.35; *d.f*. = 1,38; *P* < 0.001) and Samurai (generalised linear model with Poisson distribution: *X*^2^ = 12.50; *d.f*. = 1,38; *P* = 0.04) cultivars treated with CJ showed significant increases in *M. persicae* mortality compared to their control (blank formulation) treated counterparts.

**Figure 2:**
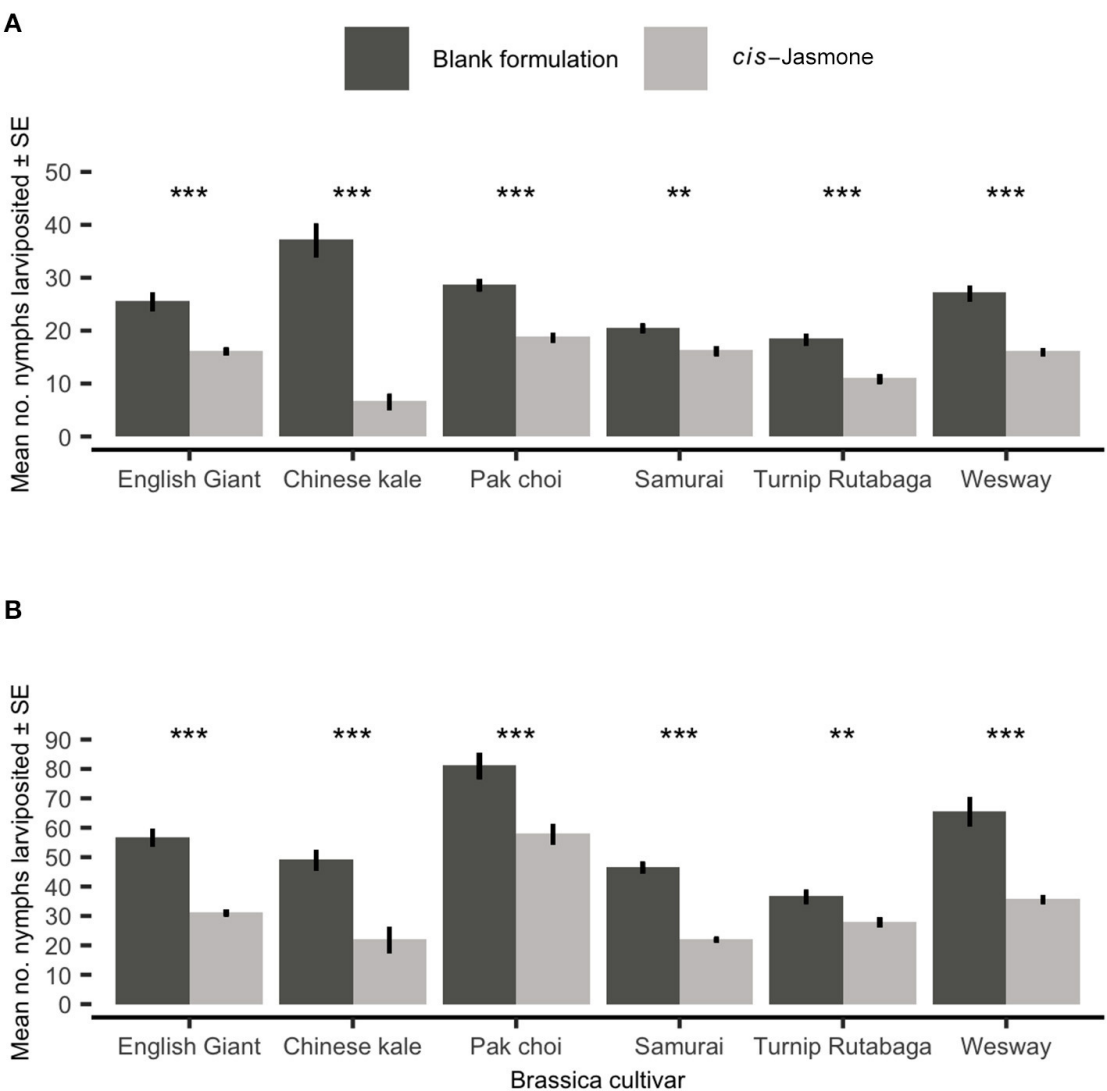
*Myzus persicae* larviposition (Mean ± SE) after **(A)** 48 h and **(B)** 96 h in clip cages on six brassica cultivars (*n* = 10) treated with *cis-*jasmone or blank formulation (control). Asterisks denote differing levels of statistical significance: ^**^ < 0.01 and ^***^ < 0.001 (generalised linear models with quasi-Poisson probability distribution).

There was a significant reduction in nymph production on CJ treated plants across both time points (Figures 2A,B). Mean larviposition on CJ treated plants of all brassica cultivars was significantly reduced after 48 hours (Figure 2A), decreasing by 46% from 26.15 on control treated plants to 14.03 on CJ treated plants. Larviposition was reduced most on Chinese kale (GLM with quasi-Poisson distribution: *F* = 76.57; *d.f*. = 1,38; *P* < 0.001; 82 % reduction in larviposition) and the least on Samurai (GLM with quasi-Poisson distribution: *F* = 33.03; *d.f*. = 1,38; *P* < 0.001; 21% reduction in larviposition). Similarly, mean larviposition on CJ treated plants of all brassica cultivars was also significantly reduced after 96 h (Figure 2B), decreasing by 41% from 55.80 on control plants to 32.65 on CJ treated plants. Larviposition was reduced most on Chinese kale (GLM with quasi-Poisson distribution: *F* = 18.31; *d.f*. = 1,38; *P* < 0.001; 55% reduction in larviposition) and the least on Turnip Rutabaga (GLM with quasi-Poisson distribution: *F* = 9.43; *d.f*. = 1,38; *P* = 0.004; 24% reduction in larviposition).

### Aphid Settlement Bioassay

**Figure 3:**
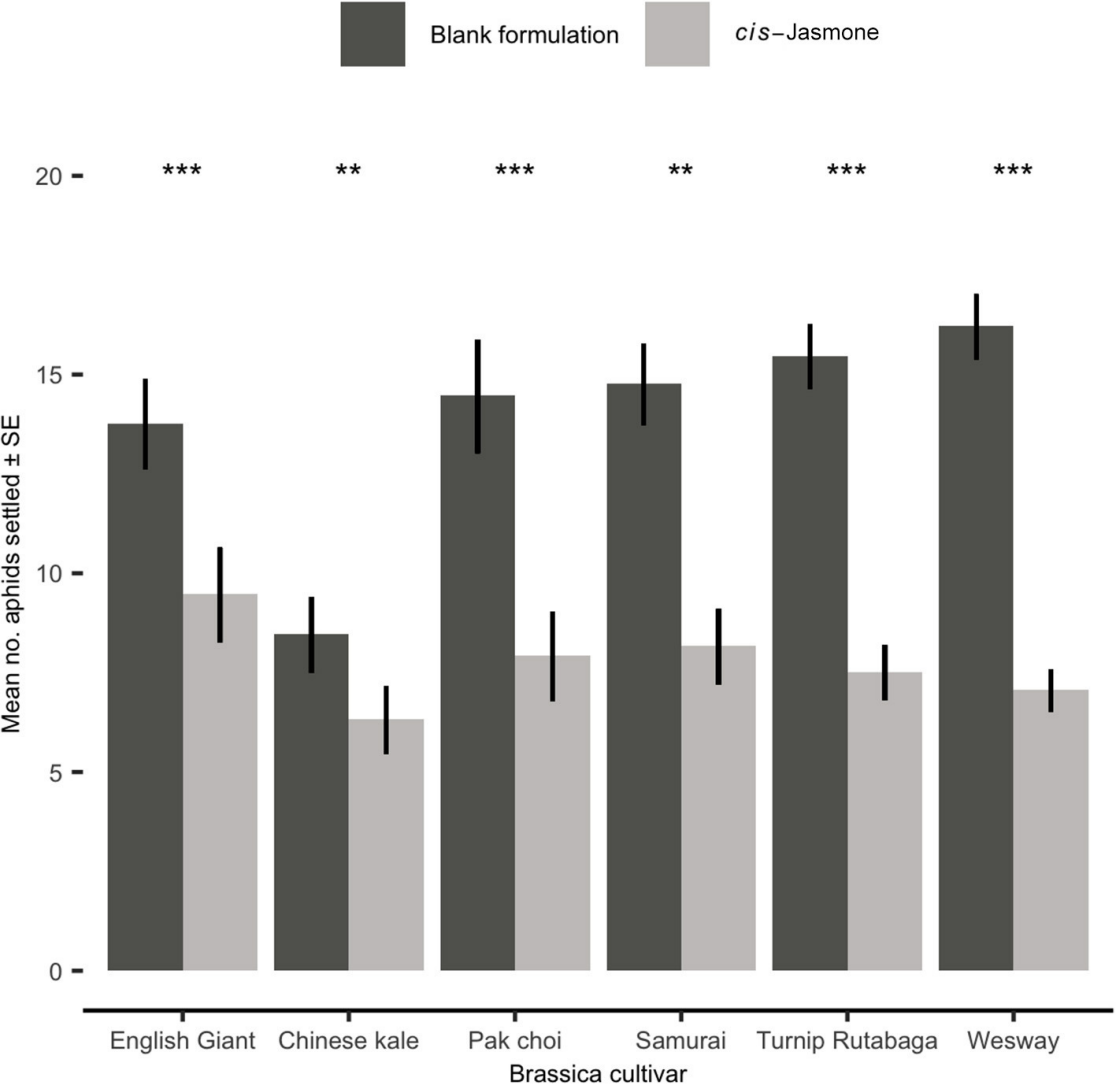
Settlement of *Myzus persicae* (Mean ± SE) after 24 h on blank formulation (control) and *cis-*jasmone treated plants in a series of choice test bioassays (50 aphids released in each replicate; *n* = 10). Asterisks denote differing levels of statistical significance: ^**^ < 0.01 and ^***^ < 0.001 (generalised linear models with either Poisson or quasi-Poisson probability distributions).

In settlement bioassays, where aphids were offered a choice between CJ treated and control plants, a clear and statistically significant reduction in aphid settlement was observed on CJ treated plants (Figure 3). This effect was consistent across all brassica cultivars tested. The preference for control over CJ treated plants was strongest for the *B. napus* cultivar Wesway, with a mean of only 7.05 aphids settling on CJ treated plants compared to 16.2 aphids settling on control treated plants (GLM with quasi-Poisson distribution: *F* = 32.39; *d.f*. = 1,38; *P* < 0.001; 30.3% of aphid settlement occurring on CJ treated plants). Similarly, aphid settlement was significantly reduced in pak choi (GLM with quasi-Poisson distribution: *F* = 98.81; *d.f*. = 1,38; *P* < 0.001), English Giant (GLM with Poisson distribution: *X*^2^ = 86.63; *d.f*. = 1,38; *P* < 0.001), Samurai (generalised linear model with Poisson distribution: *X*^2^ = 70.4: *d.f*. = 1,38; *P* < 0.001), Turnip Rutabaga (GLM with Poisson distribution: *X*^2^ = 41.12; *d.f*. = 1,38; *P* < 0.001) and Chinese kale (GLM with Poisson distribution: *X*^2^ = 86.57; *d.f*. = 1,38; *P* < 0.001). Pooling data across all cultivars tested, mean aphid settlement on CJ treated plants was 1.86 times lower than on control plants.

### Parasitoid Foraging Bioassay

**Figure 4:**
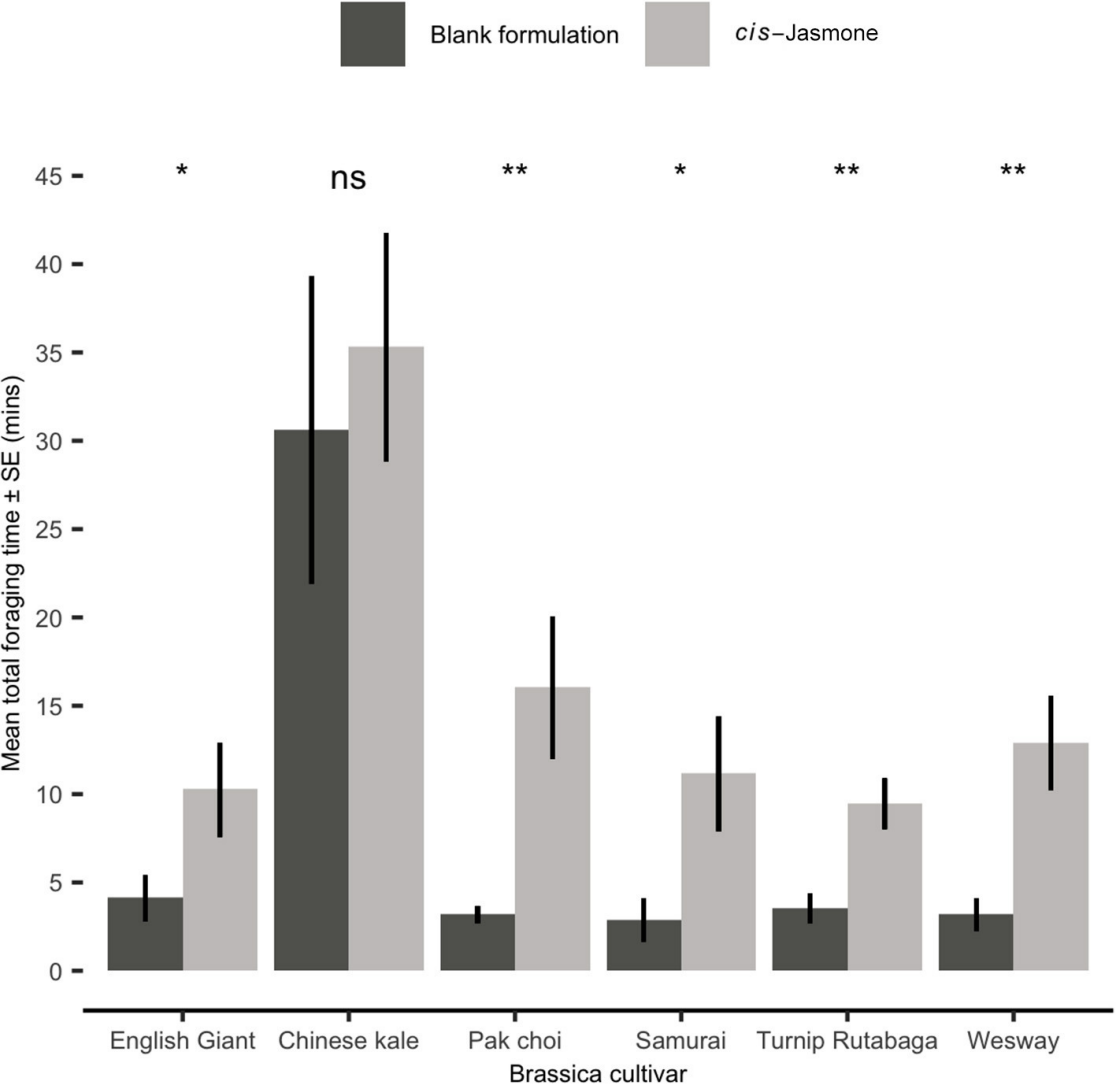
Mean total time spent foraging (Mean ± SE) by *Diaeretiella rapae* on blank formulation (control) and *cis-*jasmone treated plants (*n* = 10). Brassica cultivars capped with “ns” do not show a significant difference between control and CJ treatment while asterisks denote differing levels of statistical significance: ^*^ < 0.05 and ^**^ < 0.01 (two-sample *t*-tests).

In a foraging bioassay, parasitoid wasps spent substantially longer on CJ treated plants than on control plants (Figure 4). There was a 5.1× increase in the mean time spent on CJ treated pak choi plants (two-sample *t*-test: Welch's *t* = 3.59; *d.f*. = 10.21; *P* = 0.004), a 4.6× increase on Turnip Rutabaga (two-sample *t*-test: Welch's *t* = 3.67; *d.f*. = 17.26; *P* = 0.001), a 4.5× increase on Wesway (two-sample *t*-test: Welch's *t* = 3.89; *d.f*. = 15.48; *P* = 0.001), a 3.9× increase on Samurai (two-sample *t*-test: Welch's *t* = 2.84; *d.f*. = 14.10; *P* = 0.013), a 2.8× increase on English Giant (two-sample *t*-test: Welch's *t* = 2.22; *d.f*. = 17.39; *P* = 0.04) and no significant increase on Chinese kale (two-sample *t*-test: Welch's *t* = 0.65; *d.f*. = 16.96; *P* = 0.525).

### Parasitism Bioassay

**Figure 5:**
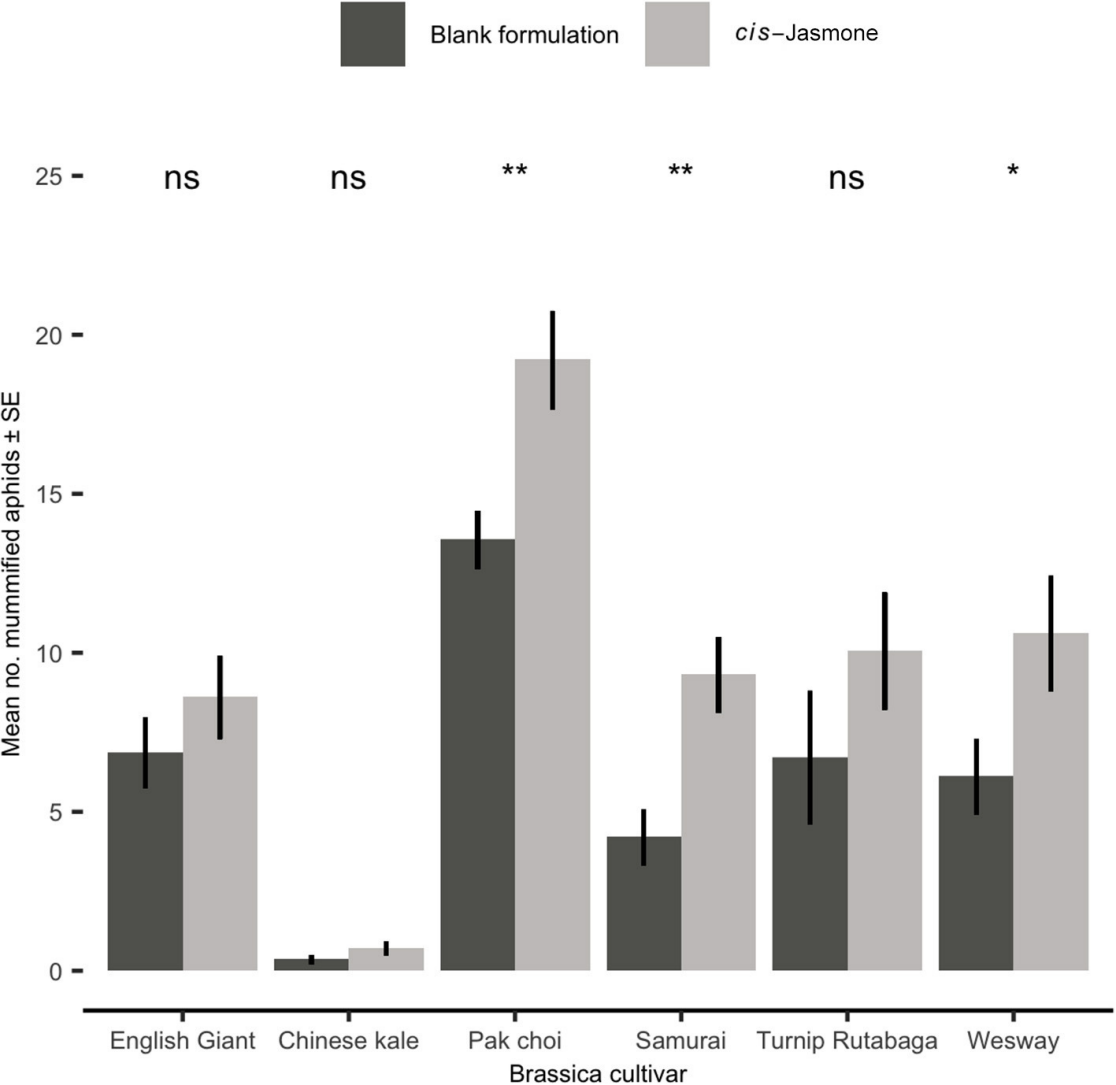
Mean number (± SE) of mummified *Myzus persicae* on blank formulation (control) and *cis-*jasmone treated plants 15 days after exposure to *Diaeretiella rapae* for 2 h (*n* = 10). Brassica cultivars capped with “ns” do not show a significant difference between control and CJ treatment while asterisks denote differing levels of statistical significance: ^*^ < 0.05 and ^**^ < 0.01 (generalised linear models with either Poisson or quasi-Poisson probability distributions).

Parasitism rates were significantly increased by CJ application in three brassica cultivars, though all six cultivars treated with CJ showed some level of increase (Figure 5). The largest increase in parasitism rates was observed on Samurai (generalised linear model with quasi-Poisson distribution: *F* = 11.74; *d.f*. = 1,38; *P* = 0.001; 121 % increase), though pak choi had the greatest total number of mummified aphids. Low levels of parasitism were observed on Chinese kale irrespective of plant treatment (Figure 5).

### Olfactometer Bioassay

#### Natural Volatile Blends

**Figure 6:**
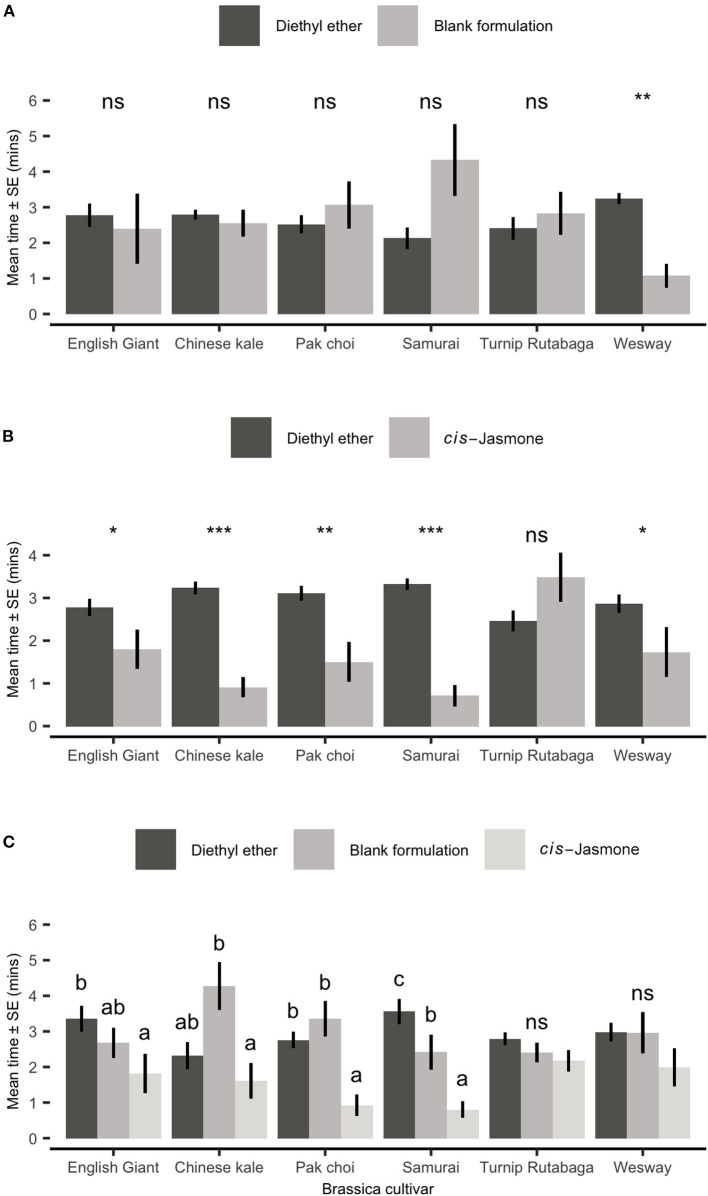
Behavioural responses of *Myzus persicae* females to volatiles from six brassica cultivars in an olfactometer bioassay. Individual aphids were given 12 min to make a choice between **(A)** one arm of blank formulation treated plants (BF—tween 80 and water) vs. three solvent diethyl ether (DEE) arms, **(B)** one arm of *cis-*jasmone treated plants (CJ) vs. three solvent control arms (DEE) and **(C)** two different treatment arms (BF, blank formulation treated plants and CJ, *cis-*jasmone treated plants) vs. two solvent arms (DEE). The shown values are mean time spent in arm ± SE (*n* = 10). For **(A,B)**, brassica cultivars capped with “ns” do not show a significant difference between plant treatment while asterisks denote differing levels of statistical significance: ^*^ < 0.05, ^**^ < 0.01 and ^***^ < 0.001 (paired *t-*test). For **(C)**, different letters above bars indicate statistically significant differences between treatments (*P* < 0.05), based on Holm-Sidak method.

When presented with a choice between a solvent control (diethyl ether; DEE) and volatiles collected from plants treated with a blank formulation, adult aphids showed no significant preference for either odour source in five of the tested cultivars (Figure 6A). Aphids did, however, spend significantly less time in the treated zone for Wesway (paired *t-*test: *t* = 4.69; *d.f*. = 9; *P* = 0.001; Figure 6A). However, when plants were CJ treated and aphids were presented with a choice of volatiles from a treated plant vs. solvent control (DEE), aphids spent significantly less time in the treated zone of the olfactometer with volatiles from CJ treated plants for 5 out of the 6 cultivars tested (Figure 6B). Turnip Rutabaga was the only cultivar in which volatiles from CJ-treated plants were not repellent. CJ-treated Chinese kale and Samurai were the most repellent. When also allowed to choose between volatiles from CJ treated plants and untreated control plants (Figure 6C), aphids spent significantly longer in the olfactometer zone with volatiles from blank formulation control plants for pak choi and the Samurai and Chinese kale cultivars.

#### Individual Synthetic Volatile Compounds

**Figure 7:**
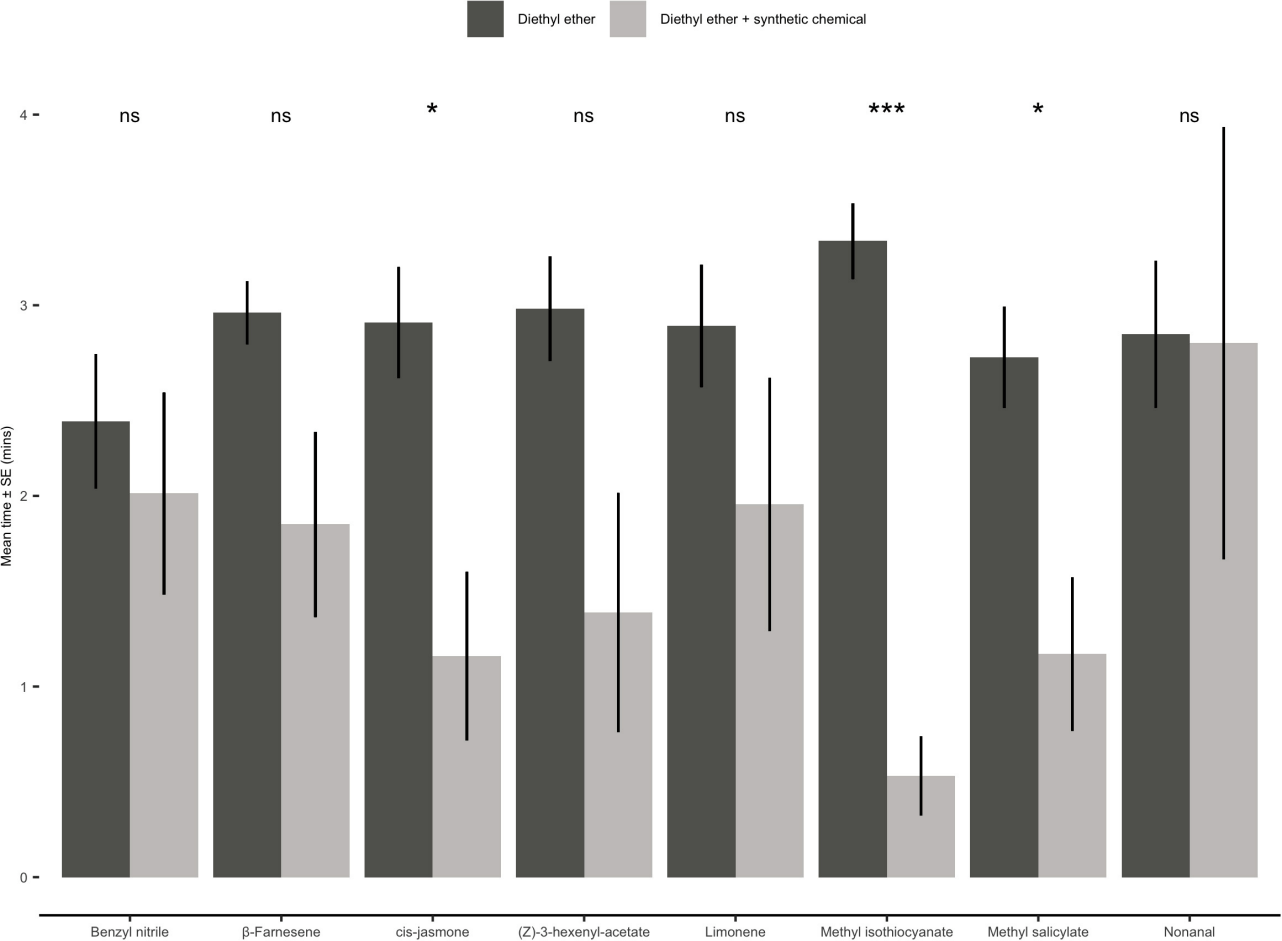
Behavioural responses of *Myzus persicae* females to eight synthetic chemical compounds. Individual aphids were given 12 min to make a choice between one arm containing a synthetic chemical compound vs. three solvent diethyl ether arms. The shown values are mean time spent in arm ± SE (*n* = 10). Synthetic chemical compounds capped with “ns” do not show a significant difference while asterisks denote differing levels of statistical significance: ^*^ < 0.05 and ^***^ < 0.001 (paired *t-*test).

When presented with a choice between a solvent control (diethyl ether) and one of the eight individual synthetic compounds, adult aphids either showed no significant preference for either odour source or avoided the olfactometer arms containing a synthetic chemical (Figure 7). Aphids spent significantly less time in the olfactometer arms containing CJ (paired *t-*test: *t* = 2.62; *d.f*. = 9; *P* = 0.03), methyl isothiocyanate (paired *t-*test: *t* = 7.86; *d.f*. = 9; *P* < 0.001) or methyl salicylate (paired *t-*test: *t* = 2.59; *d.f*. = 9; *P* = 0.03).

### Volatile Analysis

**Figure 8:**
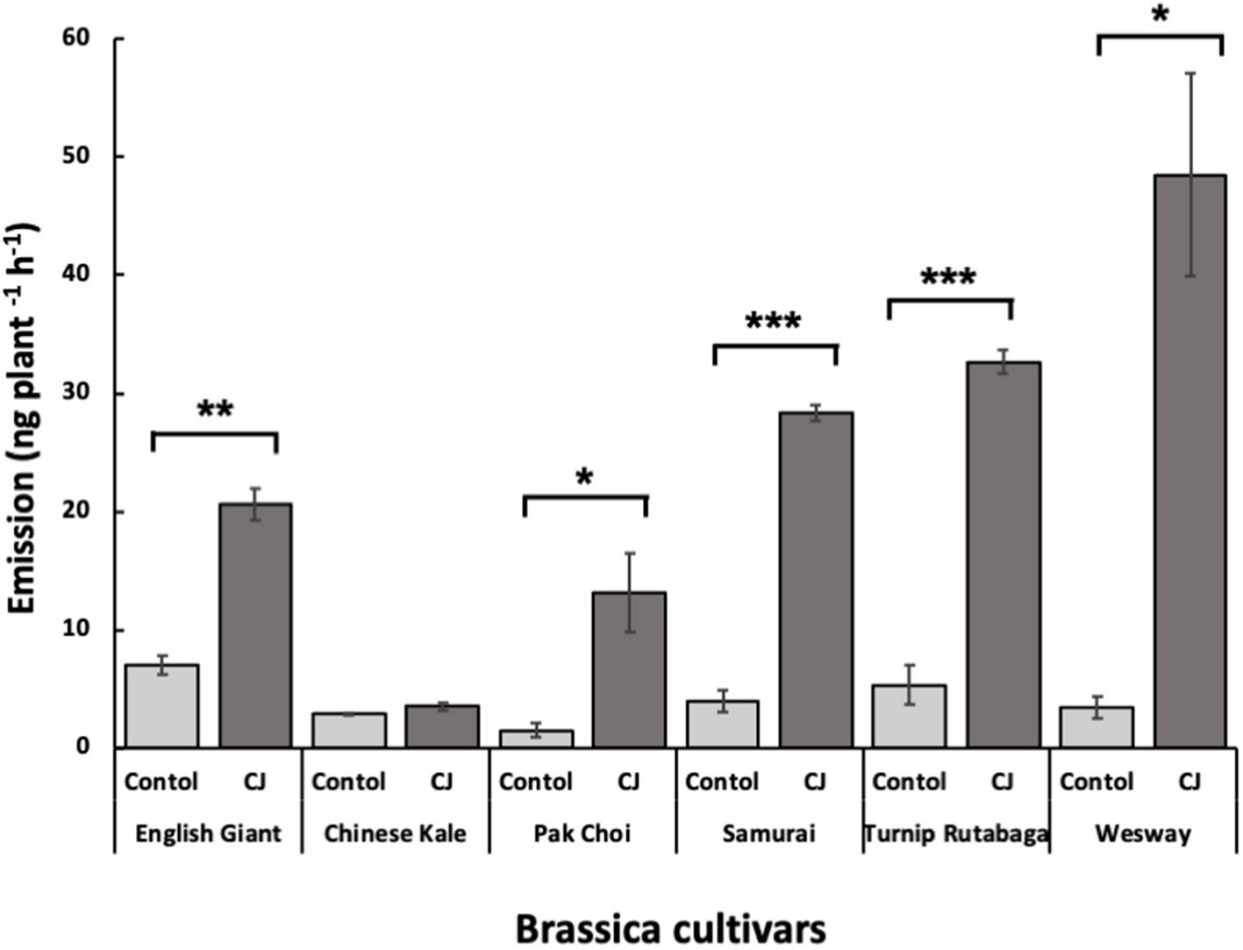
Total amount (mean nanograms plant^−1^ h^−1^ ± SE) of identified volatile organic compounds (VOCs) emitted from the six brassica cultivars with and without CJ-treatment. Asterisks indicate statistically significant differences: ^***^ < 0.001 and ^*^ < 0.05 (paired *t*-test).

**Figure 9:**
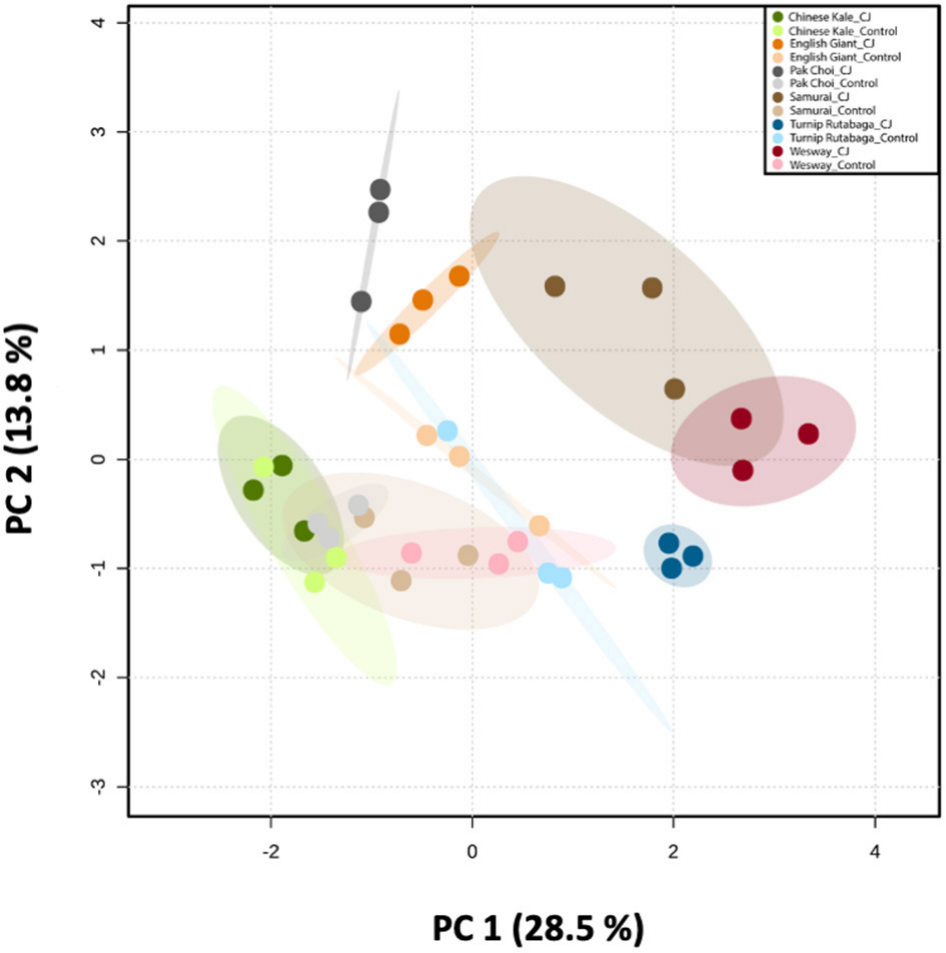
Principal Component Analysis (PCA) of the volatile compounds emitted by six brassica cultivars (i.e., Chinese kale, English Giant, pak choi, Samurai, Wesway, and Turnip Rutabaga) (*n* = 3). Score plot visualises the ordination of collected samples according to the first two PCs based on the quantitative values of emitted VOCs from different brassica cultivars with and without CJ-treatment, with the percentage of explained variation in parentheses. The ellipses show 95% confidence regions.

GC-MS analysis of headspace collections from the six studied brassica cultivars revealed 25 detectable VOCs in 8 functional classes (alcohols, aldehydes, aliphatic hydrocarbons, benzenoids, esters, ketones, N-containing compounds and terpenes). Volatile emission was found to increase with CJ treatment in all brassica cultivars except Chinese kale. There were both quantitative and qualitative changes in the volatile profile of CJ treated plants compared to control plants. Quantitative changes in total volatile emission are shown in Figure 8. Volatile emission was increased ~14-fold in Wesway, 5-fold in pak choi, 4-fold in Samurai and Turnip Rutabaga, and 2-fold in English Giant but there was no change in Chinese kale. Table 1 shows the compounds emitted. In English Giant, nonanal, (*E*)-3-tetradecene, dihydrojasmone, CJ, methyl isothiocyanate and benzyl nitrile were the main compounds induced. In Chinese kale *de novo* production of methyl isothiocyanate and benzyl nitrile was the main difference observed with CJ treatment and there was reduced emission of other compounds such as (*E*)-3-tetradecene. In pak choi there was a large increase in green leafy volatile (*Z*)-3-hexenyl acetate and 2-ethyl-1-hexanol production together with CJ, limonene, and citronellol with CJ treatment. In Samurai, induced emission of CJ itself was the main change, together with a smaller increase in emission of (*Z*)-3-hexenyl acetate and 2-ethyl-1-hexanol. In turnip rutabaga, the biggest changes were increases in (*E,E*)-α-farnesene, 2-ethyl-1-hexanol and p-cymen-7-ol with also notable induction of methyl salicylate (MeSA) and CJ. Finally, in Wesway there were marked increases in emission of 9 volatiles including CJ, 2-ethyl-1-hexanol, (*Z*)-3-hexenyl acetate and p-cymen-7-ol. Figure 9 provides a PCA analysis showing qualitative differences where the first two principal components accounted for 42.3% of the total variation in the volatile data. A clear separation based on the first principal component (PC1) is visible between CJ treated Samurai, Turnip Rutabaga, and Wesway plants, whereas another separation but based on the second principal component (PC2) is obvious for the volatile profiles of CJ treated English Giant, pak choi and Samurai plants. In descending order, the greatest loadings of PC1, were for β-elemene (0.218), *cis*-jasmone (0.213), and (*E,E*)-α-farnesene (0.170), whereas the major loadings of PC2 were for *cis*-jasmone (0.495), citronellol (0.382), and dihydrojasmone (0.257). These VOCs shown to contribute to PC1 and PC2 may impact the behaviour of *M. persicae* and *D. rapae*. A heatmap showed differential magnitude of volatile emission from the six studied brassica cultivars with and without CJ treatment (Supplementary Figure 1).

**Table 1:** Emission (in ng; mean ± SE; *n* = 3) of volatiles released by *cis*-jasmone treated (CJ) and non-treated (Control) plants ^a^.

**Plant volatile**	**KI**	**English giant**		**Chinese kale**		**Pak choi**		**Samurai**		**Turnip rutabaga**		**Wesway**	
		**Control**	**CJ**	**Control**	**CJ**	**Control**	**CJ**	**Control**	**CJ**	**Control**	**CJ**	**Control**	**CJ**
*Alcohols*													
2-ethyl-1-hexanol	1030	0.6 ± 0.1	0.7 ± 0.1	0.61 ± 0.1	0.27 ± 0.01	**0.34 ± 0.02**	**2.17 ± 0.3**	**0.51 ± 0.06**	**3.35 ± 0.4**	**0.97 ± 0.5**	**3.61 ± 0.5**	**0.36 ± 0.2**	**5.92 ± 0.9**
1-octanol	1071	ND	ND	ND	ND	ND	ND	**ND**	**0.31 ± 0.08**	**ND**	**0.37 ± 0.1**	**ND**	**0.67 ± 0.2**
2-butyl-1-octanol	1277	0.02 ± 0.01	0.6 ± 0.2	0.16 ± 0.01	0.20 ± 0.03	**ND**	**0.59 ± 0.2**	0.06 ± 0.04	0.41 ± 0.2	**0.03 ± 0.02**	**0.52 ± 0.1**	0.05 ± 0.02	1.41 ± 0.5
*Aldehydes*													
Nonanal	1104	**0.7 ± 0.2**	**3.4 ± 0.4**	ND	ND	ND	0.06 ± 0.04	**0.07 ± 0.06**	**1.15 ± 0.2**	**0.43 ± 0.2**	**1.61 ± 0.3**	**0.08 ± 0.06**	**2.62 ± 0.6**
Decanal	1206	**0.03 ± 0.01**	**0.17 ± 0.01**	ND	ND	ND	0.17 ± 0.06	0.05 ± 0.04	0.34 ± 0.1	0.22 ± 0.1	0.41 ± 0.02	0.04 ± 0.03	0.97 ± 0.2
*Aliphatic hydrocarbons*													
Dodecane	1200	0.06 ± 0.05	0.13 ± 0.03	ND	0.02 ± 0.01	0.03 ± 0.01	0.07 ± 0.05	0.04 ± 0.03	0.17 ± 0.09	0.05 ± 0.04	0.18 ± 0.07	**ND**	**0.62 ± 0.06**
*(E)*-3-tetradecene	1385	0.4 ± 0.2	1.5 ± 0.2	0.43 ± 0.06	0.24 ± 0.12	1.16 ± 0.1	1.34 ± 0.6	0.18 ± 0.07	0.92 ± 0.6	0.13 ± 0.1	0.51 ± 0.2	**ND**	**3.48 ± 0.9**
*Benzenoids*													
MeSA#	1192	ND	ND	ND	ND	ND	ND	ND	ND	**0.05 ± 0.04**	1.57 ± 0.4	ND	2.5 ± 0.6
Benzothiazole	1229	ND	ND	ND	ND	ND	0.19 ± 0.1	ND	0.77 ± 0.3	**ND**	**0.63 ± 0.1**	ND	0.39 ± 0.3
*Esters*													
*cis*-3-hexenyl acetate	1005	2.3 ± 0.3	2.9 ± 0.5	ND	ND	0.43 ± 0.3	2.81 ± 1.2	**0.91 ± 0.1**	**2.32 ± 0.2**	**1.05 ± 0.2**	**2.11 ± 0.09**	**1.28 ± 0.4**	**5.3 ± 0.8**
2-ethylhexyl acetate	1129	0.17 ± 0.01	0.22 ± 0.01	ND	ND	0.08 ± 0.03	ND	0.14 ± 0.07	0.19 ± 0.08	0.05 ± 0.03	0.19 ± 0.01	ND	ND
*Ketones*													
Dihydrojasmone	1369	0.10 ± 0.04	1.09 ± 0.6	ND	ND	**ND**	**0.27 ± 0.03**	**ND**	**0.63 ± 0.07**	ND	ND	ND	ND
*cis*-Jasmone	1394	**0.12 ± 0.06**	**1.53 ± 0.3**	**ND**	**0.11 ± 0.02**	**ND**	**1.76 ± 0.5**	**ND**	**10.9 ± 2.7**	**0.16 ± 0.13**	**1.6 ± 0.4**	**ND**	**11.7 ± 2.9**
*N-containing compounds*													
Methyl isothiocyanate	992	**0.08 ± 0.06**	**0.5 ± 0.1**	ND	0.10 ± 0.08	0.05 ± 0.04	0.09 ± 0.07	**0.09 ± 0.07**	**0.42 ± 0.01**	ND	ND	ND	0.23 ± 0.2
Benzyl nitrile	1144	**ND**	**1.7 ± 0.2**	**ND**	**0.31 ± 0.07**	0.04 ± 0.03	0.73 ± 0.24	ND	ND	**ND**	**1.26 ± 0.3**	ND	0.95 ± 0.8
*Terpenes*													
β-phellandrene	1031	ND	ND	0.18 ± 0.1	0.53 ± 0.09	ND	ND	ND	ND	ND	ND	ND	ND
D-limonene	1030	0.2 ± 0.1	0.5 ± 0.1	0.39 ± 0.06	0.55 ± 0.06	ND	1.97 ± 1.1	0.61 ± 0.4	0.56 ± 0.2	0.41 ± 0.2	1.27 ± 0.4	ND	2.17 ± 1.1
Eucalyptol	1032	ND	0.3 ± 0.1	0.75 ± 0.03	0.51 ± 0.05	ND	ND	0.07 ± 0.05	ND	ND	ND	ND	ND
Citronellol	1229	0.15 ± 0.09	0.22 ± 0.1	0.14 ± 0.1	0.31 ± 0.1	**ND**	**1.03 ± 0.2**	0.11 ± 0.08	ND	ND	ND	ND	ND
DMNT#	1116	ND	ND	ND	ND	ND	ND	ND	ND	**ND**	**0.79 ± 0.2**	ND	ND
p-cymen-7-ol	1289	0.35 ± 0.2	0.54 ± 0.08	0.15 ± 0.01	0.16 ± 0.01	**0.24 ± 0.12**	**0.85 ± 0.02**	0.32 ± 0.08	0.67 ± 0.3	0.32 ± 0.1	3.31 ± 0.5	0.23 ± 0.1	4.67 ± 1.2
α-cedrene	1411	ND	ND	ND	ND	0.06 ± 0.02	0.24 ± 0.05	ND	ND	ND	ND	ND	ND
β-elemene	1391	**1.41 ± 0.1**	**2.47 ± 0.1**	0.08 ± 0.03	0.19 ± 0.04	ND	ND	**0.75 ± 0.1**	**2.79 ± 0.4**	**0.47 ± 0.3**	**7.7 ± 1.7**	**1.23 ± 0.4**	**4.75 ± 0.5**
β-curcumene	1514	0.18 ± 0.02	0.38 ± 0.01	ND	ND	**ND**	**0.31 ± 0.07**	ND	ND	**ND**	**0.36 ± 0.1**	**ND**	**0.38 ± 0.03**
(*E, E*)-α-farnesene	1508	**0.05 ± 0.04**	**0.69 ± 0.1**	ND	ND	0.12 ± 0.1	0.31 ± 0.06	**0.03 ± 0.02**	**1.22 ± 0.03**	**0.68 ± 0.1**	**4.58 ± 0.7**	**0.18 ± 0.1**	**0.66 ± 0.06**

a *Plants were treated 24 h before the start of VOCs air entrainment. Under each chemical class, VOCs are ordered in accordance with their increasing retention time in a gas chromatograph and Kovats index. Bold values indicate significant differences between treatments (t-test; *P* < 0.05). # [DMNT: (E)-4, 8-dimethyl-1, 3, 7-nonatriene; MeSA: Methyl salicylate; ND: Not Detected]. VOCs were tentatively identified based on spectra, Kovats retention index and NIST 17 library matches. KI: Kovats index determined on the intermediately non polar HP5-MS column (https://webbook.nist.gov/; http://www.pherobase.com/)*.

## Discussion

Taken together, our data show that CJ treatment of a diverse sample of crop brassica cultivars can, as hypothesised, make the plants less suitable and less attractive for *M. persicae* aphids but more attractive to their key parasitoid, *D. rapae*. Aphid survival and nymph production were reduced on CJ treated plants and aphids avoided CJ treated plants. Conversely, *D. rapae* spent longer foraging on CJ treated plants. However, this effect was brassica cultivar dependent. This is not surprising because any induction of plant defence triggered by a chemical elicitor depends on the existence of an inducible defence trait(s) (Bruce, 2014). Evidence can be seen for differential response between brassica cultivars from the volatile analysis with the biggest changes in volatile emission in Wesway and no significant change in Chinese kale. Despite this, CJ-induced Chinese kale was the best-performing brassica in terms of reduced aphid survival on plants.

Interestingly, there appeared to be a difference between cultivars in direct and indirect defence: Chinese kale (*Brassica oleracea* cv. “Warwick L9”) was the only cultivar where adult aphid survival was substantially and significantly reduced by CJ treatment indicating that direct defence against the herbivore had increased. In contrast, CJ treatment of Chinese kale had no effect on parasitoid foraging or parasitism levels indicating that there was no effect on indirect defence via tritrophic interactions with the aphid parasitoid. In all other cultivars parasitoid foraging time was significantly increased by CJ treatment and in three of them (pak choi, Turnip Rutabaga and Wesway) a significant increase in number of aphid mummies was found.

With aphids confined in clip cages in no-choice experiments, adult survival was reduced by CJ treatment in Chinese kale at both 48 and 96 h time intervals with approximately half as many aphids surviving on the treated plants. There was also a significant reduction in adult survival with CJ treatment in Turnip Rutabaga at 96 h, but the effect size was not as large as in Chinese kale. Aphid reproduction was affected more than adult survival because a significant reduction in nymph larviposition was found with CJ treatment in all six cultivars tested, which was also the case for other crop plants treated with CJ (Dewhirst et al., 2012; Sobhy et al., 2020). A reduction in larviposition would slow down aphid population growth rates and this would be valuable in pest management because the high reproduction rate of aphids is one of the main reasons why they are such formidable pests (Leather et al., 2017).

Settlement bioassays revealed that aphid colonisation of CJ treated plants was significantly lower in all six cultivars tested. This included Chinese kale although the effect size was lower with this cultivar. Reduced aphid settlement would further slow down aphid infestations because initial populations settling on plants would also be lower with CJ treatment. Olfactometer bioassays revealed that volatiles from CJ treated plants were repellent to aphids in most cultivars. Surprisingly, no aphid repellence was observed with aphids exposed to volatiles from CJ-treated Turnip Rutabaga although volatile analysis showed a 4-fold increase in volatile emission with CJ treatment. This suggests that the chemical composition or quality matters more than the absolute quantity for aphid repellence. Also, a repellent effect was found with Chinese kale although total volatile emission was not increased by CJ-treatment. This again suggests the type of volatile emitted matters. Methyl isothiocyanate and benzyl nitrile were not detected in control Chinese kale volatiles but were found in volatiles from CJ-treated plants and it is possible these compounds could have caused the observed repellent effect but further studies would be needed to confirm this. *cis-*Jasmone itself was found in the volatiles of control English Giant and Turnip Rutabaga with increased emission after CJ treatment. It was not found in the other cultivars until after CJ treatment and the largest amounts were found in Wesway and Samurai. As CJ is highly volatile, was found in some control plants and varied considerably between varieties, we interpret this as being production of CJ by the plants themselves. The precursor dihydrojasmone was also found in English Giant, pak choi and Samurai.

A foraging bioassay was used to investigate how long parasitoids spent on treated plants after being released on them. This bioassay was used because retention of parasitoids after arrival is more important in determining parasitism levels than initial arrival rates (Budenberg et al., 1992). Significant increases in parasitoid foraging time were found in five out of the six cultivars tested. Earlier studies in *Arabidopsis* (Bruce et al., 2008) had shown that parasitoids spent approximately twice as long foraging on CJ treated plants compared to control plants. For several of the crop brassica species tested here the size of the effect was considerably bigger, for example, on pak choi and Wesway parasitoids spent 5 and 4.5 times longer on CJ treated plants than on control plants. The cultivars in which there was the biggest effect on parasitoid foraging were also the ones where the biggest increases in volatile emission were observed. This suggests that parasitoid foraging could be influenced by CJ-induced volatile emission although further experiments would be required to confirm this. A parasitism bioassay showed that numbers of aphid mummies found was significantly increased with CJ treatment in the cultivars pak choi, Turnip Rutabaga, and Wesway. CJ treatment had no effect on parasitism in Chinese kale but it is interesting to note that although parasitism on Chinese kale was low parasitoids spent an extended time foraging even on untreated Chinese kale. The most likely explanation for this is that CJ-treatment not only affected aphid survival with the direct defence effect discussed above but also made the aphids less suitable as hosts for the parasitoid.

Volatile analysis revealed both quantitative and qualitative changes in the volatile profile of CJ treated plants which is clearly illustrated by heatmap analysis. With the exception of Chinese kale, the tested brassica cultivars exhibited higher volatile emission, reached more than 4-fold in some cultivars, of key volatile compounds such as CJ, methyl isothiocyanate, benzyl nitrile, (*Z*)-3-hexenyl acetate, 2-ethyl-1-hexanol, nonanal, β-elemene, (*E,E*)-α-farnesene, and MeSA. Concordant with this, treating other crop plants with CJ induced the emission of several VOCs, including MeSA (Hegde et al., 2012; Sobhy et al., 2017, 2020), CJ (Sobhy et al., 2017, 2020), (*E*)-β-farnesene (Delaney et al., 2013; Oluwafemi et al., 2013; Sobhy et al., 2017, 2020) and (*Z*)-3-hexenyl acetate (Hegde et al., 2012; Delaney et al., 2013). These induced volatiles have been shown previously to mediate brassica/aphid/parasitoid interactions (Bruce et al., 2008; Pope et al., 2008; Verheggen et al., 2013). Thus, one possible interpretation for the notable reduction in aphid settlement and survival observed on CJ treated plants could be attributed to the higher emission of aphid defence-related herbivore-induced plant volatiles (HIPVs) following CJ application. Aphids may therefore perceive such elevated volatile emissions from CJ-treated plants as signals of a greater risk of competition from conspecifics or as a greater risk of predation from natural enemies (Bruce and Pickett, 2011). Supporting the first assumption, a reduced settlement (Cascone et al., 2015; Markovic et al., 2019), fecundity (Digilio et al., 2012; Maurya et al., 2020), and feeding behaviour (Kang et al., 2018) of several aphid species were reported when reared on plants exposed to HIPVs. The latter scenario is further supported by our parasitoid foraging and parasitism rate bioassays where parasitoid females spent substantially longer time on CJ treated plants and higher numbers of mummified aphids were recorded on CJ treated plants. Indeed, studies have previously shown that CJ treatment induce the plant indirect defence increasing their attractiveness to parasitic wasps (Bruce et al., 2008; Dewhirst et al., 2012). When individually testing the synthetics of these elevated volatiles following CJ treatment, methyl isothiocyanate, MeSA and CJ were most repellent to aphids. Indeed, these VOCs are key semiochemicals in aphid trophic interactions and are directly associated with aphid repellency and/or attraction of aphid natural enemies (Bruce et al., 2003a, 2008; Zhu and Park, 2005; Mallinger et al., 2011; Lin et al., 2016).

While there was variation between cultivars, the current results are promising because effects on aphid performance and settlement or parasitoid foraging were found in all cultivars. In five of the six cultivars tested, both aphid settlement was reduced and parasitoid foraging increased. This simultaneous “slow down” of aphid growth combined with a “speed up” of parasitoid activity could be very valuable in pest management because it could perhaps mean that natural enemies are better able to keep pace with pest population growth. Combined effects on aphids and parasitoids would be useful in an integrated pest management framework (Stenberg, 2017). The next step in development of CJ as a treatment for use against aphids in brassicas is to conduct field experiments and we have this planned for future research. We found an increase in volatile emission from CJ treated plants and this could perhaps explain why aphid settlement was reduced and parasitoid foraging increased. It would be interesting to look at the genetics underpinning these effects in future research.

## Data Availability Statement

The original contributions presented in the study are included in the article/Supplementary Material, further inquiries can be directed to the corresponding author/s.

## Author Contributions

JA, JR, IS, WK, and TB conceived the ideas and designed the experiments. JA, AC, and JR performed the experiments and collected the data. JA, JR, and IS analyzed the data. JA, JR, IS, and TB led the writing of the manuscript. All authors contributed critically to the drafts and gave final approval for publication.

## Funding

The research was funded by BBSRC project BB/R021708/1. JA was supported by the Government of India.

## Conflict of Interest

The authors declare that the research was conducted in the absence of any commercial or financial relationships that could be construed as a potential conflict of interest.

## Publisher's Note

All claims expressed in this article are solely those of the authors and do not necessarily represent those of their affiliated organizations, or those of the publisher, the editors and the reviewers. Any product that may be evaluated in this article, or claim that may be made by its manufacturer, is not guaranteed or endorsed by the publisher.
