# Supplementary material for: Effects of cis-Jasmone Treatment of Brassicas on Interactions With Myzus persicae Aphids and Their Parasitoid Diaeretiella rapae
Source: Front Plant Sci. 2021 Oct 1;12:711896. doi: 10.3389/fpls.2021.711896 (PMC8517453; doi:10.3389/fpls.2021.711896)
Supplement: Supplementary file 1 [file Data_Sheet_1.docx]

Supplementary information:

Effects of *cis*-jasmone treatment of brassicas on interactions with *Myzus persicae* aphids and their parasitoid *Diaeretiella rapae*

Jamin Ali^1^, Anca Covaci^1^, Joe M Roberts^2^, Islam S Sobhy^1,3^, William DJ Kirk^1^, Toby JA Bruce^1*^

^1^School of Life Sciences, Keele University, Keele, Staffordshire, ST5 5BG, UK

^2^Centre for Integrated Pest Management, Agriculture and Environment Department, Harper Adams University, Newport, Shropshire, TF10 8NB, UK

^3^Department of Plant Protection, Faculty of Agriculture, Suez Canal University, 41522 Ismailia, Egypt

*** Correspondence:**Toby JA Bruce
t.j.a.bruce@keele.ac.uk

Keywords: induced defence, aphid, tritrophic interactions, biological control, crop protection.

Frontiers in Plant Science - Research Topic "*Inducing Plant Resistance Against Insects Using Exogenous Bioactive Chemicals: Key Advances and Future Perspectives*"


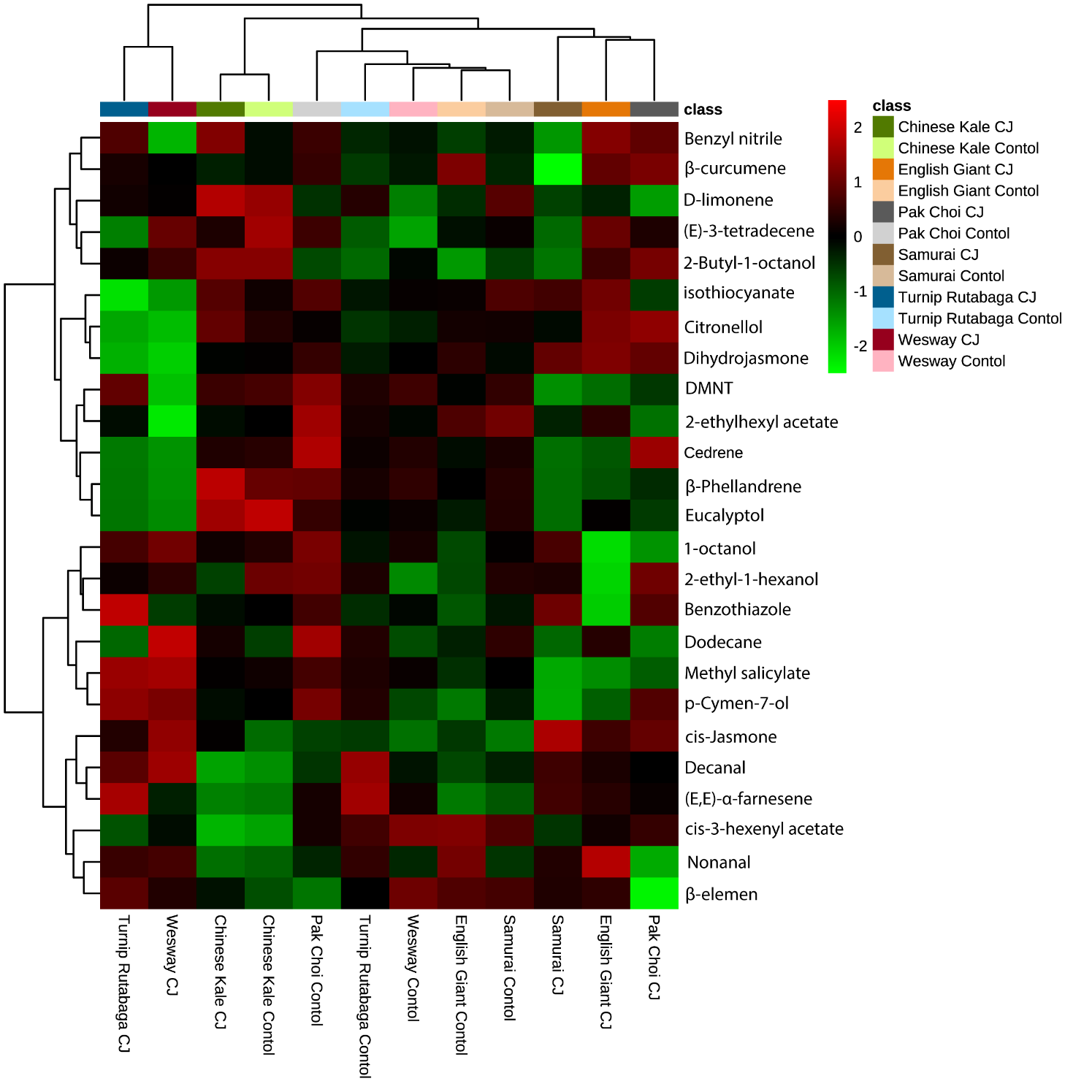


**FIGURE S1** Hierarchical cluster of the volatile profiles emitted by six brassica cultivars (i.e. Chinese kale, English Giant, pak choi, Samurai, Wesway and Turnip Rutabaga). Log transformed values of biological replicates (n = 3) are represented as a heatmap according to the scale in which red corresponds to higher VOCs emission whereas green denotes lower VOCs emission. The sample/VOC grouping is based on Ward clustering algorithm of the Euclidean distance measure for the differentially emitted VOCs detected by ANOVA (*P* < 0.05). Quantitative data are shown in Table 1.
